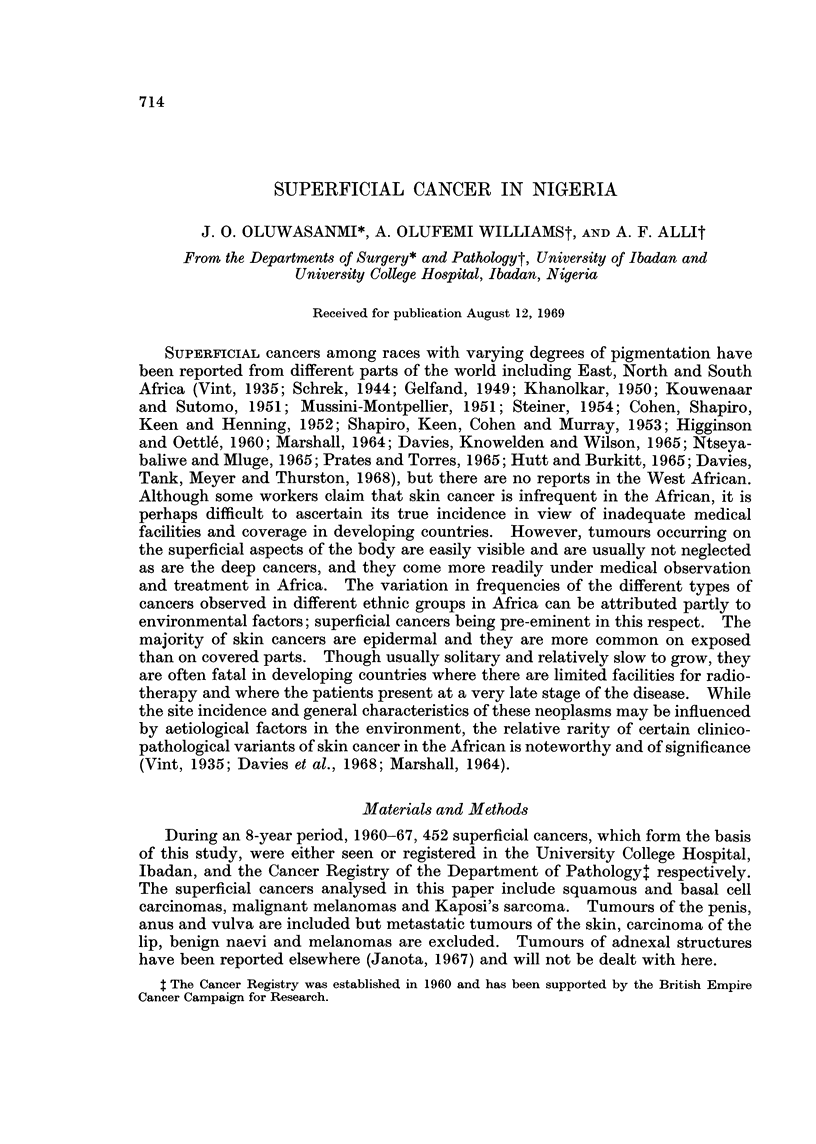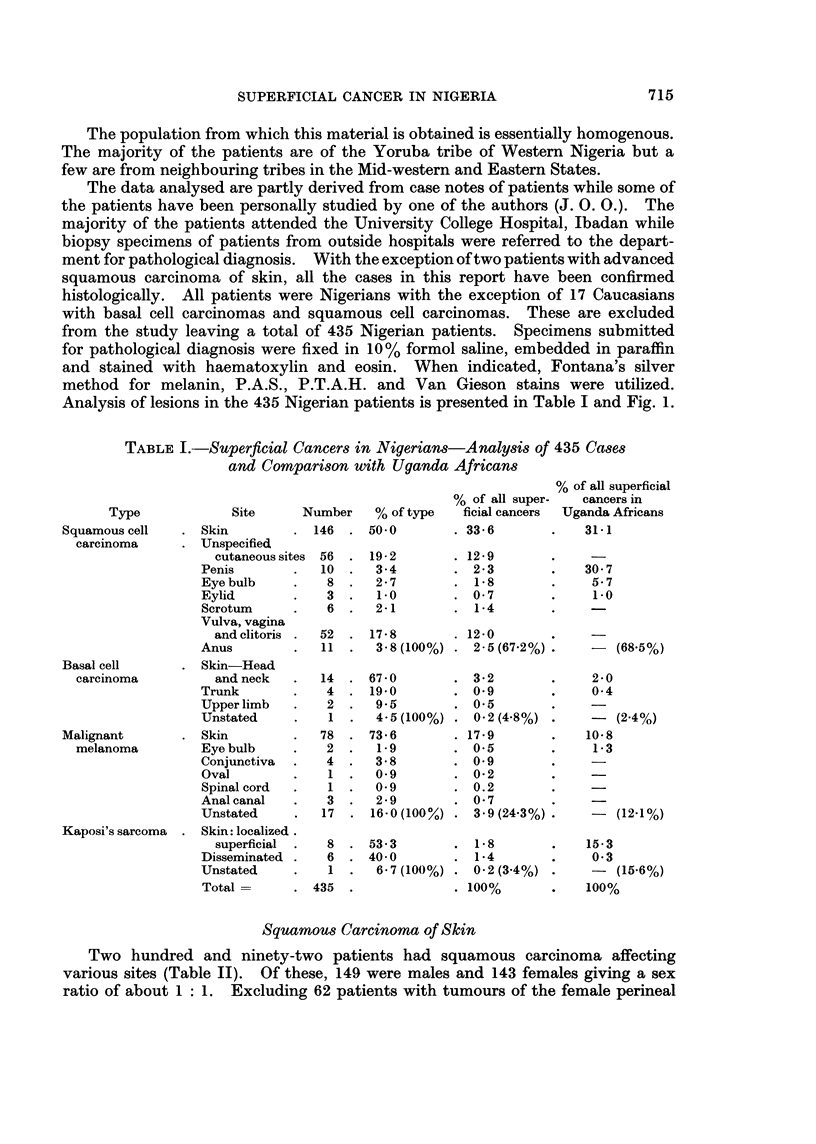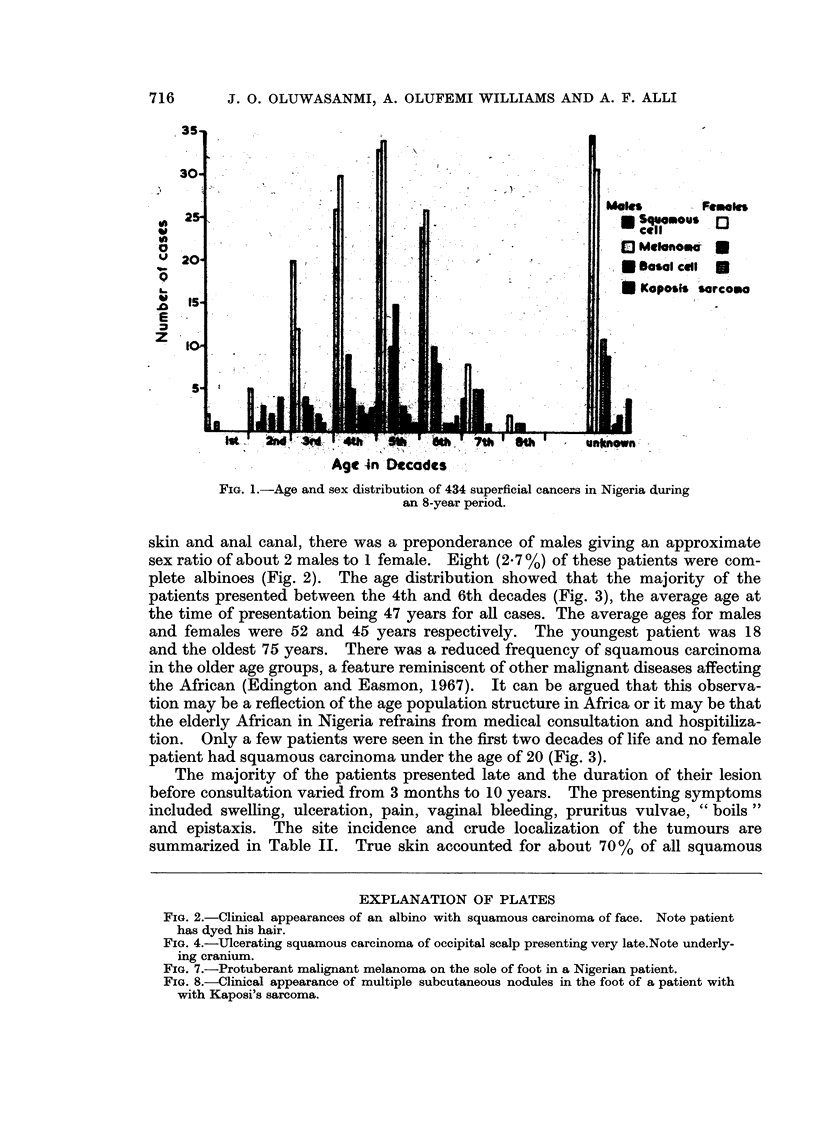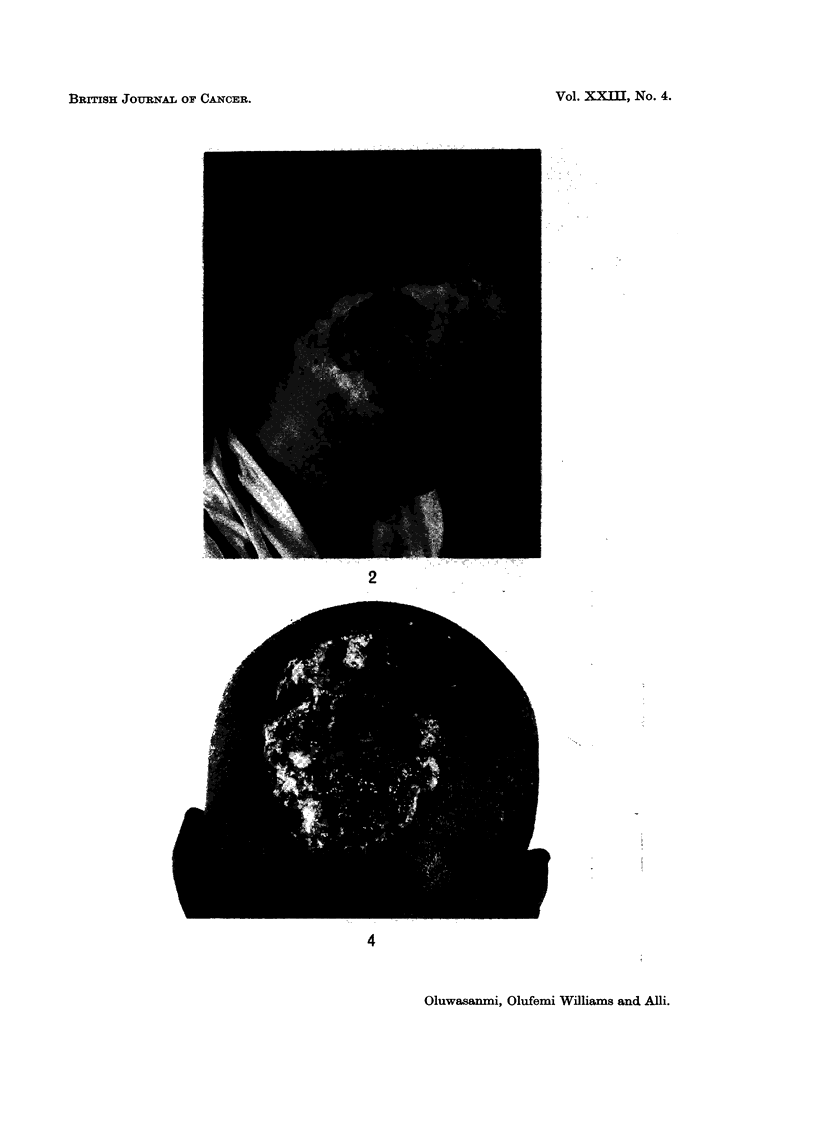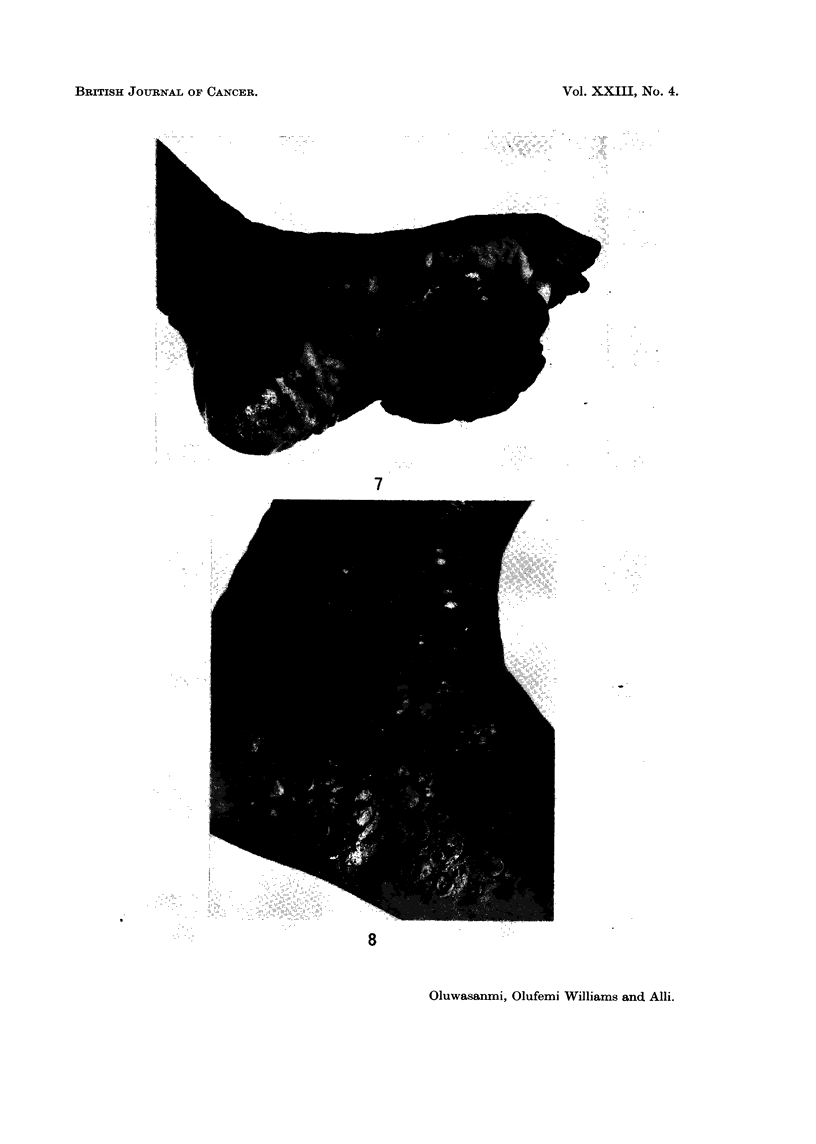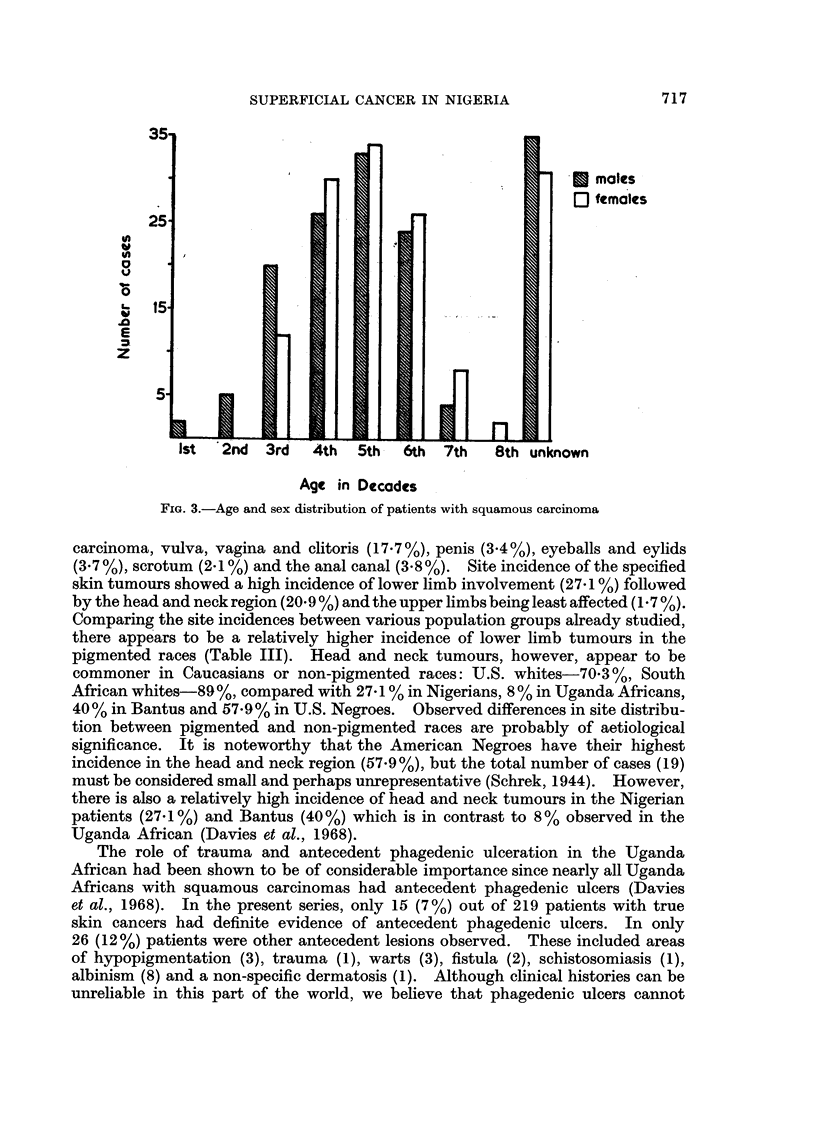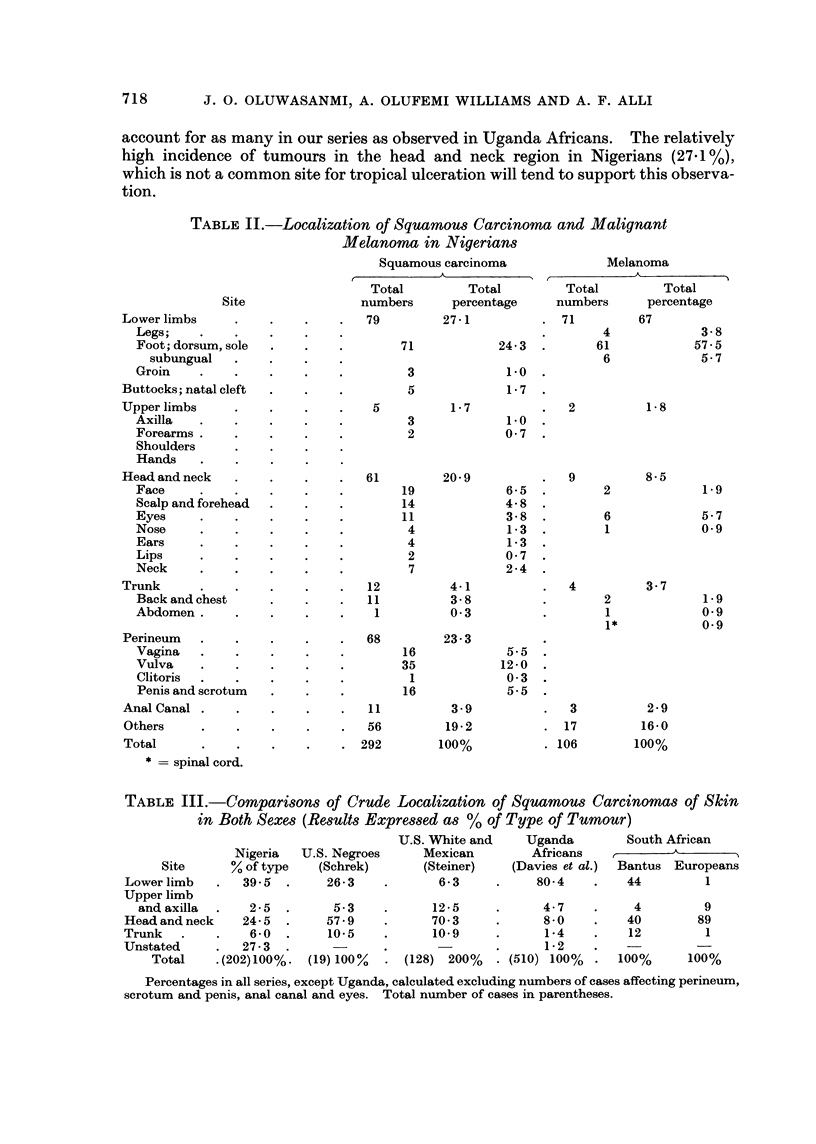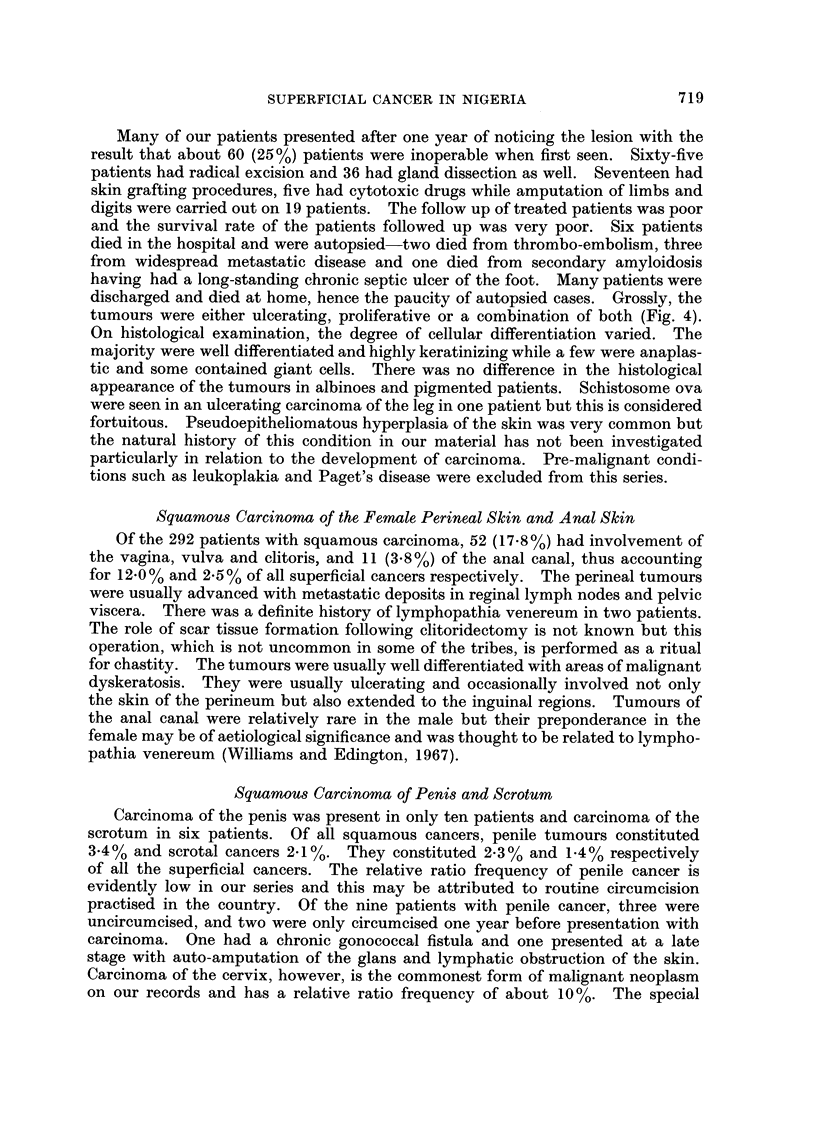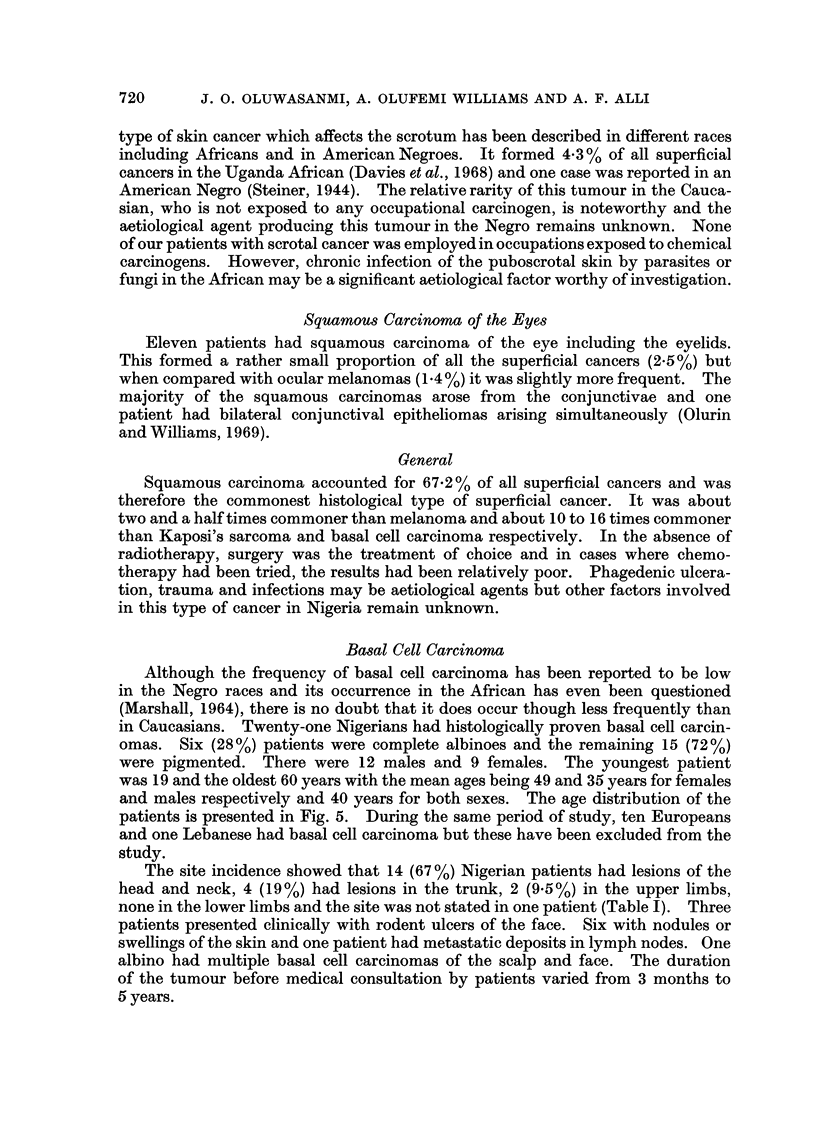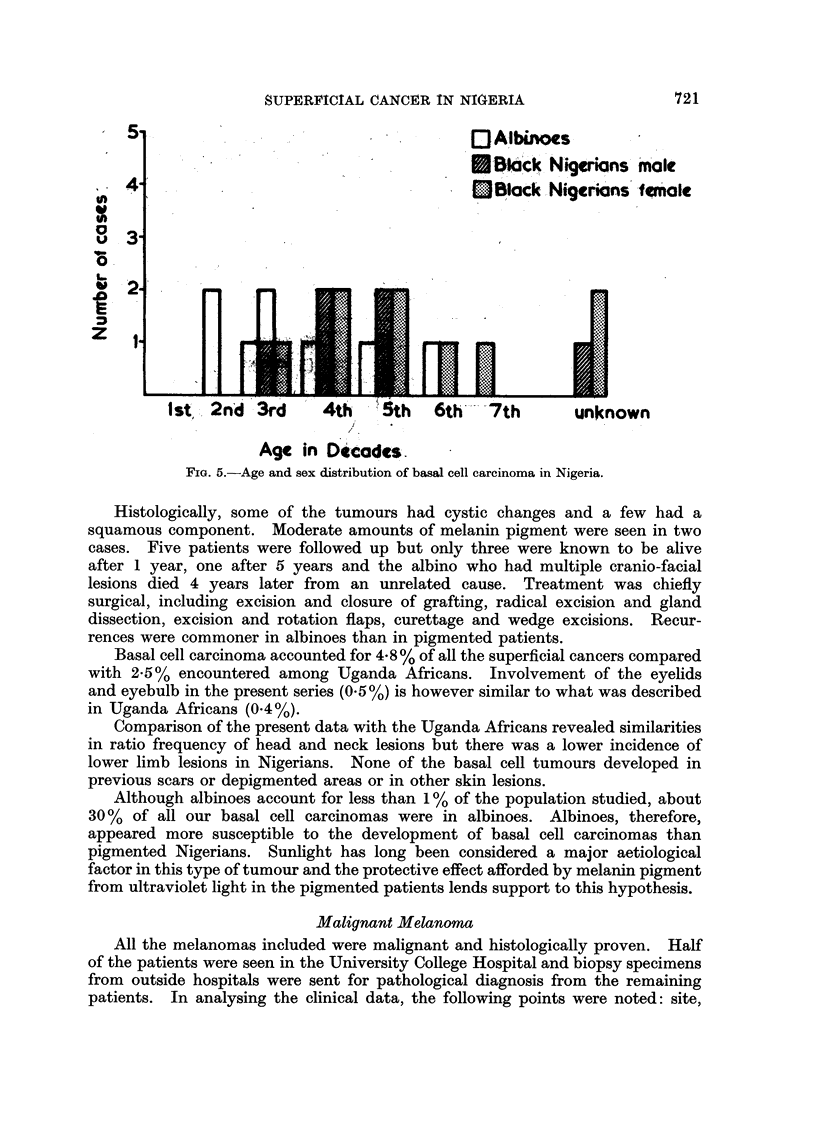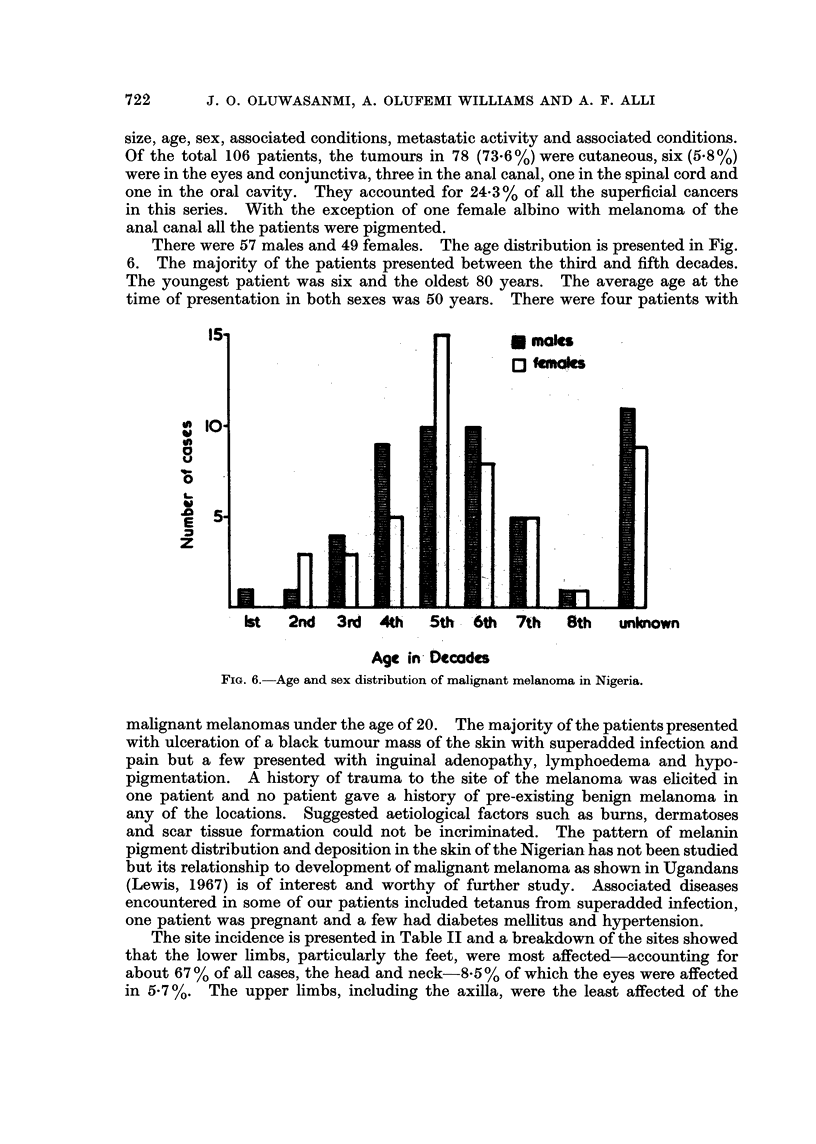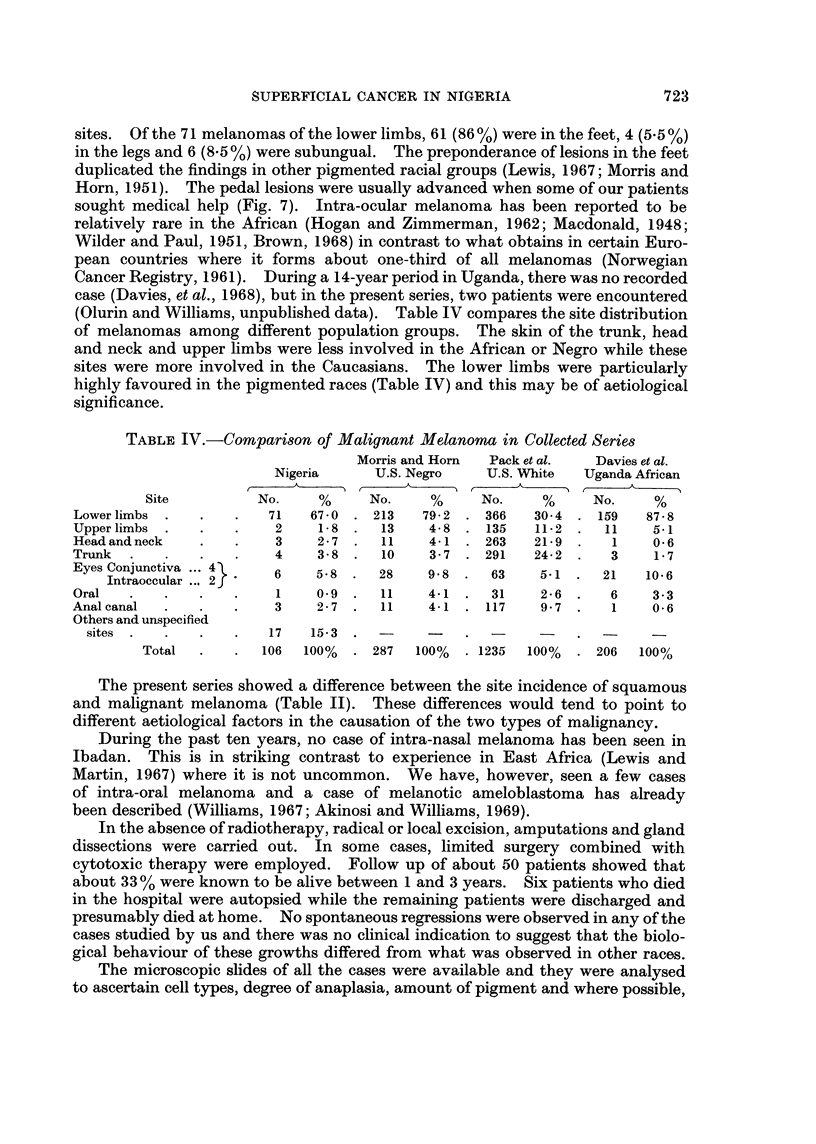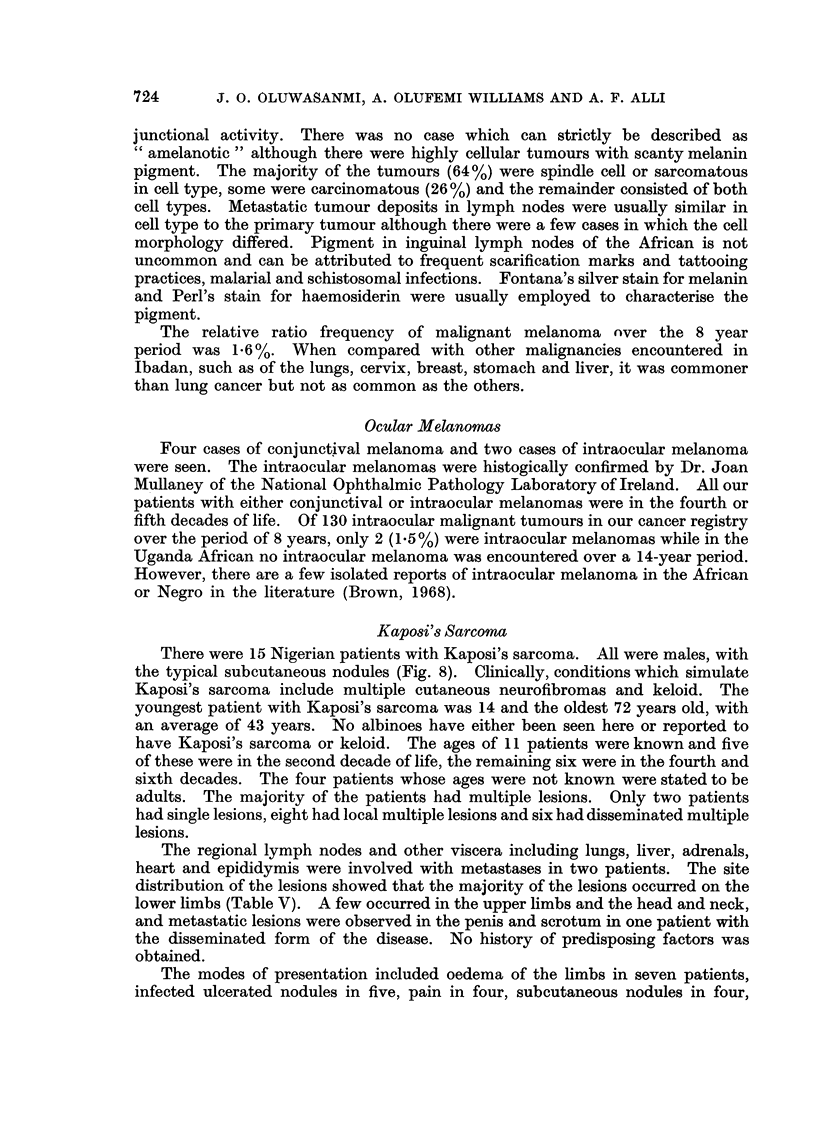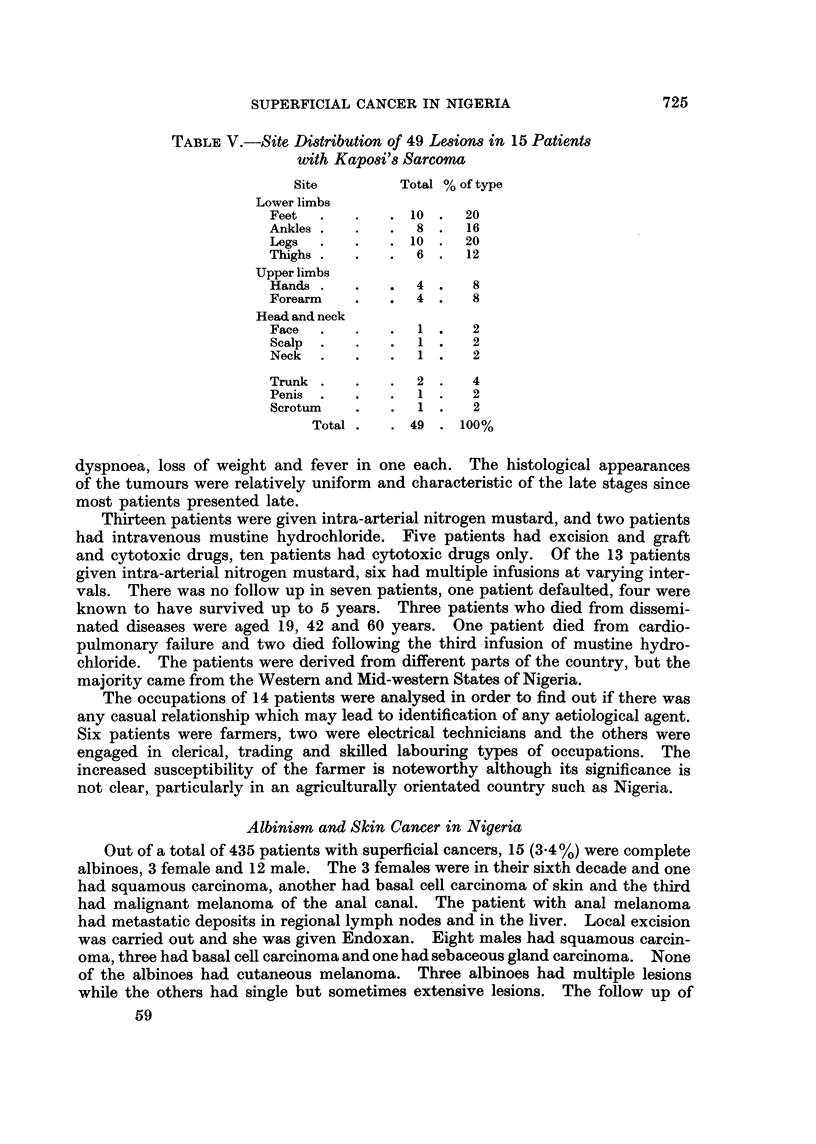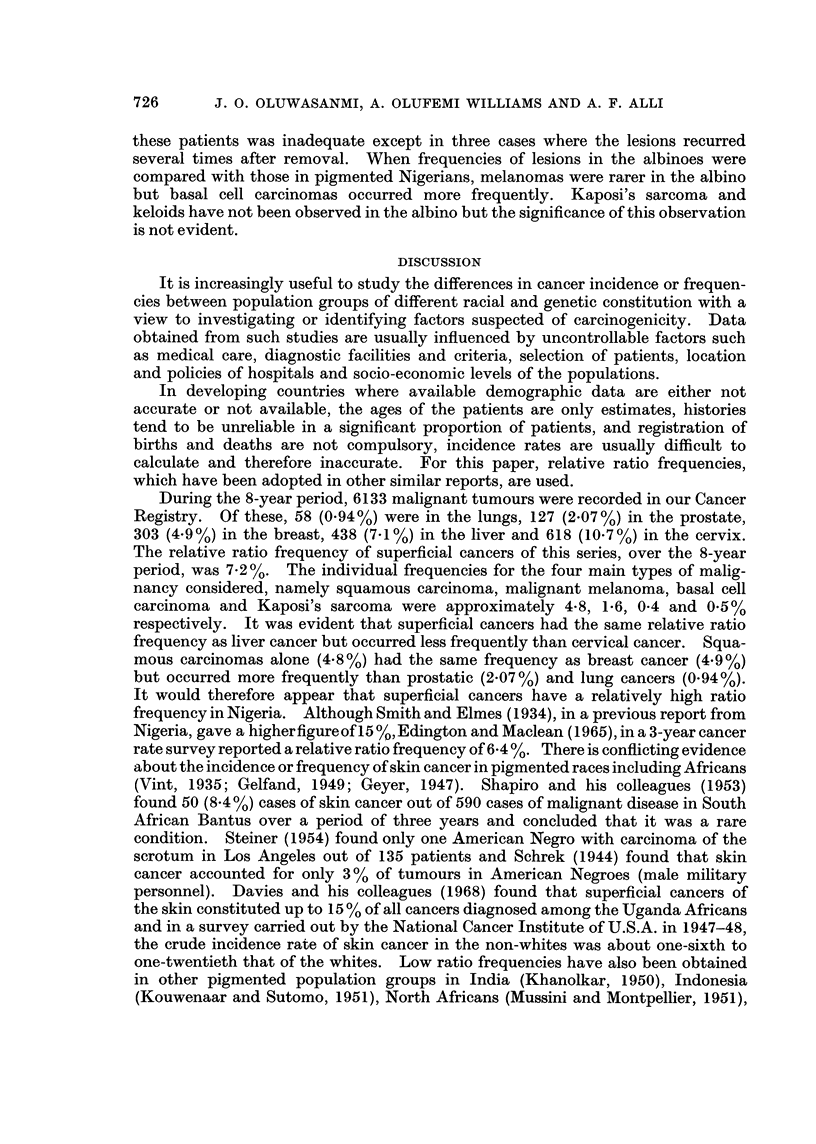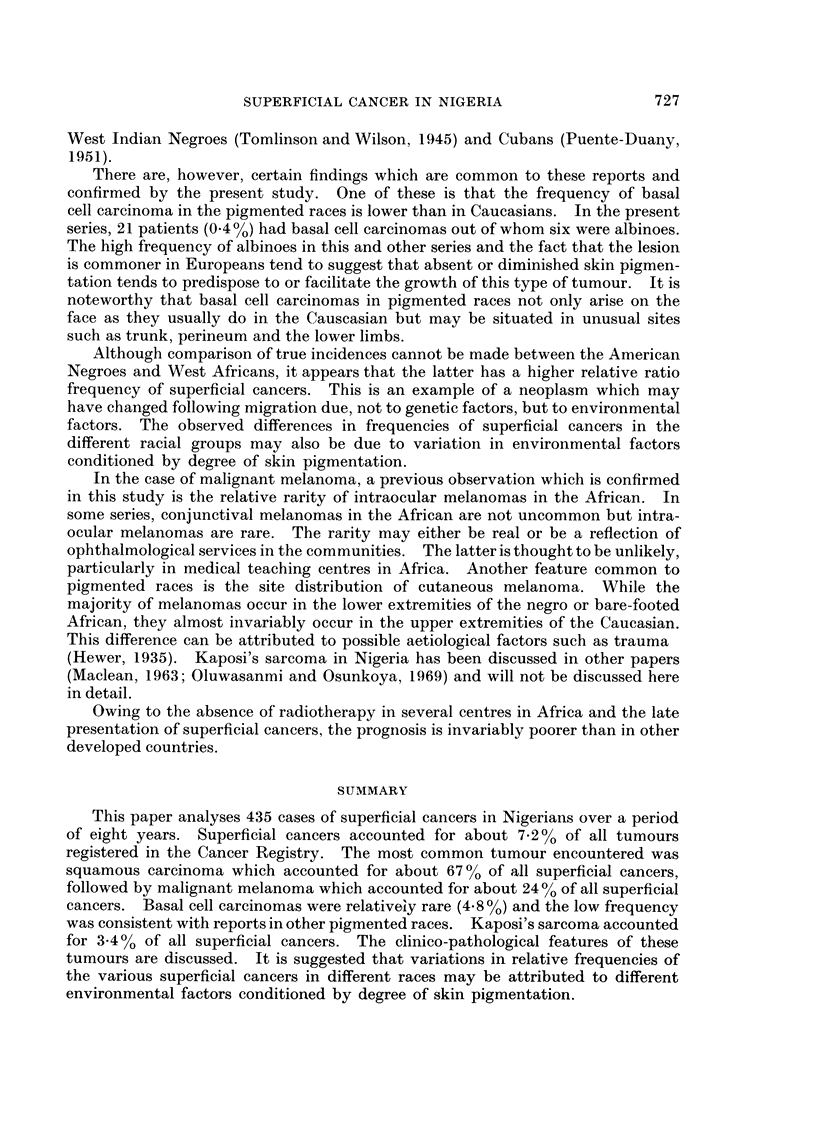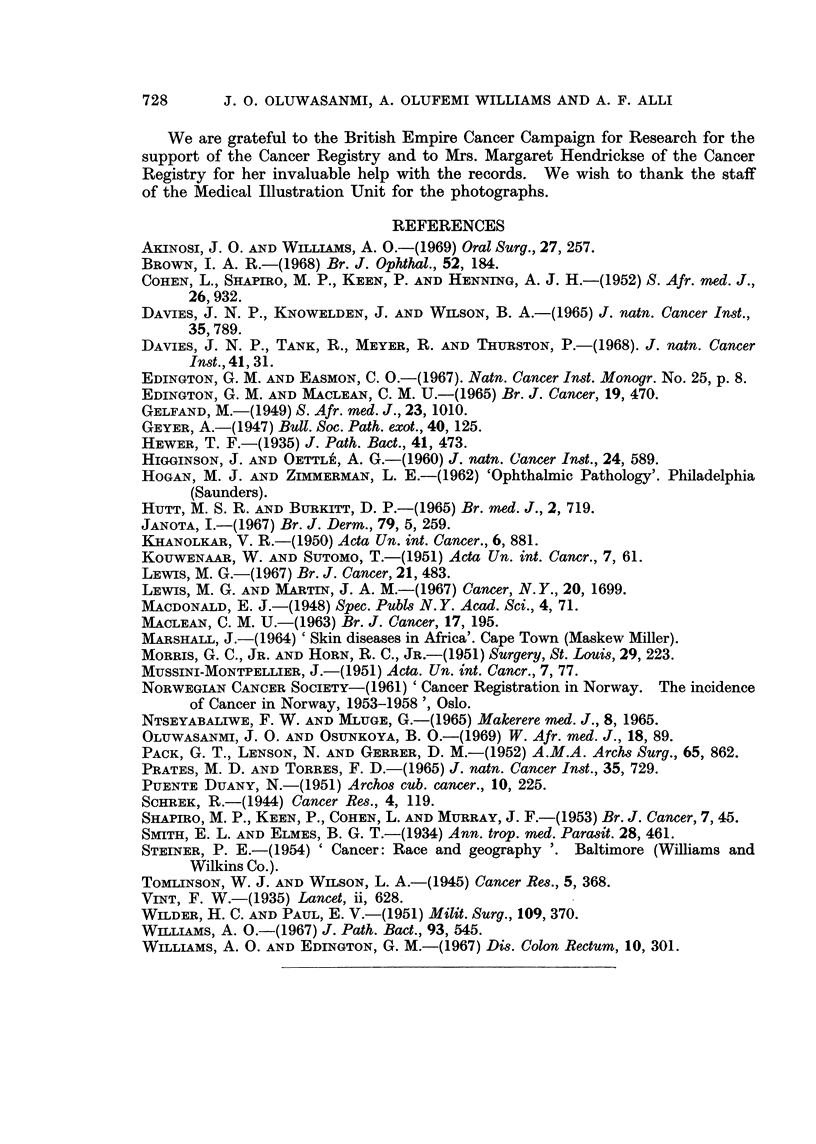# Superficial cancer in Nigeria.

**DOI:** 10.1038/bjc.1969.88

**Published:** 1969-12

**Authors:** J. O. Oluwasanmi, A. O. Williams, A. F. Alli

## Abstract

**Images:**


### SUPERFICIAL CANCER IN NIGERIA

J. O. OLUWASANMI*, A. OLUFEMI WILLIAMS†, AND A. F. ALLI†

*From the Departments of Surgery* and Pathology†, University of Ibadan and University College Hospital, Ibadan, Nigeria*

Received for publication August 12, 1969

SUPERFICIAL cancers among races with varying degrees of pigmentation have been reported from different parts of the world including East, North and South Africa (Vint, 1935; Schrek, 1944; Gelfand, 1949; Khanolkar, 1950; Kouwenaar and Sutomo, 1951; Mussini-Montpellier, 1951; Steiner, 1954; Cohen, Shapiro, Keen and Henning, 1952; Shapiro, Keen, Cohen and Murray, 1953; Higginson and Oettlé, 1960; Marshall, 1964; Davies, Knowelden and Wilson, 1965; Ntseya-baliwe and Mluge, 1965; Prates and Torres, 1965; Hutt and Burkitt, 1965; Davies, Tank, Meyer and Thurston, 1968), but there are no reports in the West African. Although some workers claim that skin cancer is infrequent in the African, it is perhaps difficult to ascertain its true incidence in view of inadequate medical facilities and coverage in developing countries. However, tumours occurring on the superficial aspects of the body are easily visible and are usually not neglected as are the deep cancers, and they come more readily under medical observation and treatment in Africa. The variation in frequencies of the different types of cancers observed in different ethnic groups in Africa can be attributed partly to environmental factors; superficial cancers being pre-eminent in this respect. The majority of skin cancers are epidermal and they are more common on exposed than on covered parts. Though usually solitary and relatively slow to grow, they are often fatal in developing countries where there are limited facilities for radio-therapy and where the patients present at a very late stage of the disease. While the site incidence and general characteristics of these neoplasms may be influenced by aetiological factors in the environment, the relative rarity of certain clinico-pathological variants of skin cancer in the African is noteworthy and of significance (Vint, 1935; Davies *et al.*, 1968; Marshall, 1964).

### *Materials and Methods*

During an 8-year period, 1960–67, 452 superficial cancers, which form the basis of this study, were either seen or registered in the University College Hospital, Ibadan, and the Cancer Registry of the Department of Pathology‡ respectively. The superficial cancers analysed in this paper include squamous and basal cell carcinomas, malignant melanomas and Kaposi's sarcoma. Tumours of the penis, anus and vulva are included but metastatic tumours of the skin, carcinoma of the lip, benign naevi and melanomas are excluded. Tumours of adnexal structures have been reported elsewhere (Janota, 1967) and will not be dealt with here.

‡ The Cancer Registry was established in 1960 and has been supported by the British Empire Cancer Campaign for Research.

The population from which this material is obtained is essentially homogenous. The majority of the patients are of the Yoruba tribe of Western Nigeria but a few are from neighbouring tribes in the Mid-western and Eastern States.

The data analysed are partly derived from case notes of patients while some of the patients have been personally studied by one of the authors (J. O. O.). The majority of the patients attended the University College Hospital, Ibadan while biopsy specimens of patients from outside hospitals were referred to the department for pathological diagnosis. With the exception of two patients with advanced squamous carcinoma of skin, all the cases in this report have been confirmed histologically. All patients were Nigerians with the exception of 17 Caucasians with basal cell carcinomas and squamous cell carcinomas. These are excluded from the study leaving a total of 435 Nigerian patients. Specimens submitted for pathological diagnosis were fixed in 10% formol saline, embedded in paraffin and stained with haematoxylin and eosin. When indicated, Fontana's silver method for melanin, P.A.S., P.T.A.H. and Van Gieson stains were utilized. Analysis of lesions in the 435 Nigerian patients is presented in Table I and Fig. 1.

TABLE I.—*Superficial Cancers in Nigerians—Analysis of* 435 *Cases and Comparison with Uganda Africans*

| Type | Site | Number | % of type | % of all superficial cancers | % of all superficial cancers in Uganda Africans |
|---|---|---|---|---|---|
| Squamous cell carcinoma | Skin | 146 | 50·0 | 33·6 | 31·1 |
| | Unspecified cutaneous sites | 56 | 19·2 | 12·9 | — |
| | Penis | 10 | 3·4 | 2·3 | 30·7 |
| | Eye bulb | 8 | 2·7 | 1·8 | 5·7 |
| | Eylid | 3 | 1·0 | 0·7 | 1·0 |
| | Scrotum | 6 | 2·1 | 1·4 | — |
| | Vulva, vagina and clitoris | 52 | 17·8 | 12·0 | — |
| | Anus | 11 | 3·8 (100%) | 2·5 (67·2%) | — (68·5%) |
| Basal cell carcinoma | Skin—Head and neck | 14 | 67·0 | 3·2 | 2·0 |
| | Trunk | 4 | 19·0 | 0·9 | 0·4 |
| | Upper limb | 2 | 9·5 | 0·5 | — |
| | Unstated | 1 | 4·5 (100%) | 0·2 (4·8%) | — (2·4%) |
| Malignant melanoma | Skin | 78 | 73·6 | 17·9 | 10·8 |
| | Eye bulb | 2 | 1·9 | 0·5 | 1·3 |
| | Conjunctiva | 4 | 3·8 | 0·9 | — |
| | Oval | 1 | 0·9 | 0·2 | — |
| | Spinal cord | 1 | 0·9 | 0.2 | — |
| | Anal canal | 3 | 2·9 | 0·7 | — |
| | Unstated | 17 | 16·0 (100%) | 3·9 (24·3%) | — (12·1%) |
| Kaposi's sarcoma | Skin: localized superficial | 8 | 53·3 | 1·8 | 15·3 |
| | Disseminated | 6 | 40·0 | 1·4 | 0·3 |
| | Unstated | 1 | 6·7 (100%) | 0·2 (3·4%) | — (15·6%) |
| | Total = | 435 | | 100% | 100% |

### *Squamous Carcinoma of Skin*

Two hundred and ninety-two patients had squamous carcinoma affecting various sites (Table II). Of these, 149 were males and 143 females giving a sex ratio of about 1 : 1. Excluding 62 patients with tumours of the female perineal

FIG. 1.—Age and sex distribution of 434 superficial cancers in Nigeria during an 8-year period.

skin and anal canal, there was a preponderance of males giving an approximate sex ratio of about 2 males to 1 female. Eight (2·7%) of these patients were complete albinoes (Fig. 2). The age distribution showed that the majority of the patients presented between the 4th and 6th decades (Fig. 3), the average age at the time of presentation being 47 years for all cases. The average ages for males and females were 52 and 45 years respectively. The youngest patient was 18 and the oldest 75 years. There was a reduced frequency of squamous carcinoma in the older age groups, a feature reminiscent of other malignant diseases affecting the African (Edington and Easmon, 1967). It can be argued that this observation may be a reflection of the age population structure in Africa or it may be that the elderly African in Nigeria refrains from medical consultation and hospitilization. Only a few patients were seen in the first two decades of life and no female patient had squamous carcinoma under the age of 20 (Fig. 3).

The majority of the patients presented late and the duration of their lesion before consultation varied from 3 months to 10 years. The presenting symptoms included swelling, ulceration, pain, vaginal bleeding, pruritus vulvae, " boils " and epistaxis. The site incidence and crude localization of the tumours are summarized in Table II. True skin accounted for about 70% of all squamous

---

EXPLANATION OF PLATES

FIG. 2.—Clinical appearances of an albino with squamous carcinoma of face. Note patient has dyed his hair.

FIG. 4.—Ulcerating squamous carcinoma of occipital scalp presenting very late.Note underlying cranium.

FIG. 7.—Protuberant malignant melanoma on the sole of foot in a Nigerian patient.

FIG. 8.—Clinical appearance of multiple subcutaneous nodules in the foot of a patient with with Kaposi's sarcoma.

2

4

Oluwasanmi, Olufemi Williams and Alli.

7

8

Oluwasanmi, Olufemi Williams and Alli.

FIG. 3.—Age and sex distribution of patients with squamous carcinoma carcinoma, vulva, vagina and clitoris (17·7%), penis (3·4%), eyeballs and eylids (3·7%), scrotum (2·1%) and the anal canal (3·8%). Site incidence of the specified skin tumours showed a high incidence of lower limb involvement (27·1%) followed by the head and neck region (20·9%) and the upper limbs being least affected (1·7%). Comparing the site incidences between various population groups already studied, there appears to be a relatively higher incidence of lower limb tumours in the pigmented races (Table III). Head and neck tumours, however, appear to be commoner in Caucasians or non-pigmented races: U.S. whites—70·3%, South African whites—89%, compared with 27·1% in Nigerians, 8% in Uganda Africans, 40% in Bantus and 57·9% in U.S. Negroes. Observed differences in site distribution between pigmented and non-pigmented races are probably of aetiological significance. It is noteworthy that the American Negroes have their highest incidence in the head and neck region (57·9%), but the total number of cases (19) must be considered small and perhaps unrepresentative (Schrek, 1944). However, there is also a relatively high incidence of head and neck tumours in the Nigerian patients (27·1%) and Bantus (40%) which is in contrast to 8% observed in the Uganda African (Davies *et al.*, 1968).

The role of trauma and antecedent phagedenic ulceration in the Uganda African had been shown to be of considerable importance since nearly all Uganda Africans with squamous carcinomas had antecedent phagedenic ulcers (Davies *et al.*, 1968). In the present series, only 15 (7%) out of 219 patients with true skin cancers had definite evidence of antecedent phagedenic ulcers. In only 26 (12%) patients were other antecedent lesions observed. These included areas of hypopigmentation (3), trauma (1), warts (3), fistula (2), schistosomiasis (1), albinism (8) and a non-specific dermatosis (1). Although clinical histories can be unreliable in this part of the world, we believe that phagedenic ulcers cannot account for as many in our series as observed in Uganda Africans. The relatively high incidence of tumours in the head and neck region in Nigerians (27·1%), which is not a common site for tropical ulceration will tend to support this observation.

TABLE II.—*Localization of Squamous Carcinoma and Malignant Melanoma in Nigerians*

| Site | Squamous carcinoma: Total numbers | | Squamous carcinoma: Total percentage | | Melanoma: Total numbers | | Melanoma: Total percentage | |
|---|---|---|---|---|---|---|---|---|
| Lower limbs | 79 | | 27·1 | | 71 | | 67 | |
| Legs; | | | | | | 4 | | 3·8 |
| Foot; dorsum, sole | | 71 | | 24·3 | | 61 | | 57·5 |
| subungual | | | | | | 6 | | 5·7 |
| Groin | | 3 | | 1·0 | | | | |
| Buttocks; natal cleft | | 5 | | 1·7 | | | | |
| Upper limbs | 5 | | 1·7 | | 2 | | 1·8 | |
| Axilla | | 3 | | 1·0 | | | | |
| Forearms | | 2 | | 0·7 | | | | |
| Shoulders | | | | | | | | |
| Hands | | | | | | | | |
| Head and neck | 61 | | 20·9 | | 9 | | 8·5 | |
| Face | | 19 | | 6·5 | | 2 | | 1·9 |
| Scalp and forehead | | 14 | | 4·8 | | | | |
| Eyes | | 11 | | 3·8 | | 6 | | 5·7 |
| Nose | | 4 | | 1·3 | | 1 | | 0·9 |
| Ears | | 4 | | 1·3 | | | | |
| Lips | | 2 | | 0·7 | | | | |
| Neck | | 7 | | 2·4 | | | | |
| Trunk | 12 | | 4·1 | | 4 | | 3·7 | |
| Back and chest | 11 | | 3·8 | | | 2 | | 1·9 |
| Abdomen | 1 | | 0·3 | | | 1 | | 0·9 |
| | | | | | | 1* | | 0·9 |
| Perineum | 68 | | 23·3 | | | | | |
| Vagina | | 16 | | 5·5 | | | | |
| Vulva | | 35 | | 12·0 | | | | |
| Clitoris | | 1 | | 0·3 | | | | |
| Penis and scrotum | | 16 | | 5·5 | | | | |
| Anal Canal | 11 | | 3·9 | | 3 | | 2·9 | |
| Others | 56 | | 19·2 | | 17 | | 16·0 | |
| Total | 292 | | 100% | | 106 | | 100% | |

* = spinal cord.

TABLE III.—*Comparisons of Crude Localization of Squamous Carcinomas of Skin in Both Sexes (Results Expressed as % of Type of Tumour)*

| Site | Nigeria % of type | U.S. Negroes (Schrek) | U.S. White and Mexican (Steiner) | Uganda Africans (Davies *et al.*) | South African: Bantus | South African: Europeans |
|---|---|---|---|---|---|---|
| Lower limb | 39·5 | 26·3 | 6·3 | 80·4 | 44 | 1 |
| Upper limb and axilla | 2·5 | 5·3 | 12·5 | 4·7 | 4 | 9 |
| Head and neck | 24·5 | 57·9 | 70·3 | 8·0 | 40 | 89 |
| Trunk | 6·0 | 10·5 | 10·9 | 1·4 | 12 | 1 |
| Unstated | 27·3 | — | — | 1·2 | — | — |
| Total | (202)100% | (19) 100% | (128) 200% | (510) 100% | 100% | 100% |

Percentages in all series, except Uganda, calculated excluding numbers of cases affecting perineum, scrotum and penis, anal canal and eyes. Total number of cases in parentheses.

Many of our patients presented after one year of noticing the lesion with the result that about 60 (25%) patients were inoperable when first seen. Sixty-five patients had radical excision and 36 had gland dissection as well. Seventeen had skin grafting procedures, five had cytotoxic drugs while amputation of limbs and digits were carried out on 19 patients. The follow up of treated patients was poor and the survival rate of the patients followed up was very poor. Six patients died in the hospital and were autopsied—two died from thrombo-embolism, three from widespread metastatic disease and one died from secondary amyloidosis having had a long-standing chronic septic ulcer of the foot. Many patients were discharged and died at home, hence the paucity of autopsied cases. Grossly, the tumours were either ulcerating, proliferative or a combination of both (Fig. 4). On histological examination, the degree of cellular differentiation varied. The majority were well differentiated and highly keratinizing while a few were anaplastic and some contained giant cells. There was no difference in the histological appearance of the tumours in albinoes and pigmented patients. Schistosome ova were seen in an ulcerating carcinoma of the leg in one patient but this is considered fortuitous. Pseudoepitheliomatous hyperplasia of the skin was very common but the natural history of this condition in our material has not been investigated particularly in relation to the development of carcinoma. Pre-malignant conditions such as leukoplakia and Paget's disease were excluded from this series.

*Squamous Carcinoma of the Female Perineal Skin and Anal Skin*

Of the 292 patients with squamous carcinoma, 52 (17·8%) had involvement of the vagina, vulva and clitoris, and 11 (3·8%) of the anal canal, thus accounting for 12·0% and 2·5% of all superficial cancers respectively. The perineal tumours were usually advanced with metastatic deposits in reginal lymph nodes and pelvic viscera. There was a definite history of lymphopathia venereum in two patients. The role of scar tissue formation following clitoridectomy is not known but this operation, which is not uncommon in some of the tribes, is performed as a ritual for chastity. The tumours were usually well differentiated with areas of malignant dyskeratosis. They were usually ulcerating and occasionally involved not only the skin of the perineum but also extended to the inguinal regions. Tumours of the anal canal were relatively rare in the male but their preponderance in the female may be of aetiological significance and was thought to be related to lymphopathia venereum (Williams and Edington, 1967).

*Squamous Carcinoma of Penis and Scrotum*

Carcinoma of the penis was present in only ten patients and carcinoma of the scrotum in six patients. Of all squamous cancers, penile tumours constituted 3·4% and scrotal cancers 2·1%. They constituted 2·3% and 1·4% respectively of all the superficial cancers. The relative ratio frequency of penile cancer is evidently low in our series and this may be attributed to routine circumcision practised in the country. Of the nine patients with penile cancer, three were uncircumcised, and two were only circumcised one year before presentation with carcinoma. One had a chronic gonococcal fistula and one presented at a late stage with auto-amputation of the glans and lymphatic obstruction of the skin. Carcinoma of the cervix, however, is the commonest form of malignant neoplasm on our records and has a relative ratio frequency of about 10%. The special type of skin cancer which affects the scrotum has been described in different races including Africans and in American Negroes. It formed 4·3% of all superficial cancers in the Uganda African (Davies *et al.*, 1968) and one case was reported in an American Negro (Steiner, 1944). The relative rarity of this tumour in the Caucasian, who is not exposed to any occupational carcinogen, is noteworthy and the aetiological agent producing this tumour in the Negro remains unknown. None of our patients with scrotal cancer was employed in occupations exposed to chemical carcinogens. However, chronic infection of the puboscrotal skin by parasites or fungi in the African may be a significant aetiological factor worthy of investigation.

### *Squamous Carcinoma of the Eyes*

Eleven patients had squamous carcinoma of the eye including the eyelids. This formed a rather small proportion of all the superficial cancers (2·5%) but when compared with ocular melanomas (1·4%) it was slightly more frequent. The majority of the squamous carcinomas arose from the conjunctivae and one patient had bilateral conjunctival epitheliomas arising simultaneously (Olurin and Williams, 1969).

### *General*

Squamous carcinoma accounted for 67·2% of all superficial cancers and was therefore the commonest histological type of superficial cancer. It was about two and a half times commoner than melanoma and about 10 to 16 times commoner than Kaposi's sarcoma and basal cell carcinoma respectively. In the absence of radiotherapy, surgery was the treatment of choice and in cases where chemotherapy had been tried, the results had been relatively poor. Phagedenic ulceration, trauma and infections may be aetiological agents but other factors involved in this type of cancer in Nigeria remain unknown.

### *Basal Cell Carcinoma*

Although the frequency of basal cell carcinoma has been reported to be low in the Negro races and its occurrence in the African has even been questioned (Marshall, 1964), there is no doubt that it does occur though less frequently than in Caucasians. Twenty-one Nigerians had histologically proven basal cell carcinomas. Six (28%) patients were complete albinoes and the remaining 15 (72%) were pigmented. There were 12 males and 9 females. The youngest patient was 19 and the oldest 60 years with the mean ages being 49 and 35 years for females and males respectively and 40 years for both sexes. The age distribution of the patients is presented in Fig. 5. During the same period of study, ten Europeans and one Lebanese had basal cell carcinoma but these have been excluded from the study.

The site incidence showed that 14 (67%) Nigerian patients had lesions of the head and neck, 4 (19%) had lesions in the trunk, 2 (9·5%) in the upper limbs, none in the lower limbs and the site was not stated in one patient (Table I). Three patients presented clinically with rodent ulcers of the face. Six with nodules or swellings of the skin and one patient had metastatic deposits in lymph nodes. One albino had multiple basal cell carcinomas of the scalp and face. The duration of the tumour before medical consultation by patients varied from 3 months to 5 years.

FIG. 5.—Age and sex distribution of basal cell carcinoma in Nigeria.

Histologically, some of the tumours had cystic changes and a few had a squamous component. Moderate amounts of melanin pigment were seen in two cases. Five patients were followed up but only three were known to be alive after 1 year, one after 5 years and the albino who had multiple cranio-facial lesions died 4 years later from an unrelated cause. Treatment was chiefly surgical, including excision and closure of grafting, radical excision and gland dissection, excision and rotation flaps, curettage and wedge excisions. Recurrences were commoner in albinoes than in pigmented patients.

Basal cell carcinoma accounted for 4·8% of all the superficial cancers compared with 2·5% encountered among Uganda Africans. Involvement of the eyelids and eyebulb in the present series (0·5%) is however similar to what was described in Uganda Africans (0·4%).

Comparison of the present data with the Uganda Africans revealed similarities in ratio frequency of head and neck lesions but there was a lower incidence of lower limb lesions in Nigerians. None of the basal cell tumours developed in previous scars or depigmented areas or in other skin lesions.

Although albinoes account for less than 1% of the population studied, about 30% of all our basal cell carcinomas were in albinoes. Albinoes, therefore, appeared more susceptible to the development of basal cell carcinomas than pigmented Nigerians. Sunlight has long been considered a major aetiological factor in this type of tumour and the protective effect afforded by melanin pigment from ultraviolet light in the pigmented patients lends support to this hypothesis.

### *Malignant Melanoma*

All the melanomas included were malignant and histologically proven. Half of the patients were seen in the University College Hospital and biopsy specimens from outside hospitals were sent for pathological diagnosis from the remaining patients. In analysing the clinical data, the following points were noted: site, size, age, sex, associated conditions, metastatic activity and associated conditions. Of the total 106 patients, the tumours in 78 (73·6%) were cutaneous, six (5·8%) were in the eyes and conjunctiva, three in the anal canal, one in the spinal cord and one in the oral cavity. They accounted for 24·3% of all the superficial cancers in this series. With the exception of one female albino with melanoma of the anal canal all the patients were pigmented.

There were 57 males and 49 females. The age distribution is presented in Fig. 6. The majority of the patients presented between the third and fifth decades. The youngest patient was six and the oldest 80 years. The average age at the time of presentation in both sexes was 50 years. There were four patients with

FIG. 6.—Age and sex distribution of malignant melanoma in Nigeria.

malignant melanomas under the age of 20. The majority of the patients presented with ulceration of a black tumour mass of the skin with superadded infection and pain but a few presented with inguinal adenopathy, lymphoedema and hypopigmentation. A history of trauma to the site of the melanoma was elicited in one patient and no patient gave a history of pre-existing benign melanoma in any of the locations. Suggested aetiological factors such as burns, dermatoses and scar tissue formation could not be incriminated. The pattern of melanin pigment distribution and deposition in the skin of the Nigerian has not been studied but its relationship to development of malignant melanoma as shown in Ugandans (Lewis, 1967) is of interest and worthy of further study. Associated diseases encountered in some of our patients included tetanus from superadded infection, one patient was pregnant and a few had diabetes mellitus and hypertension.

The site incidence is presented in Table II and a breakdown of the sites showed that the lower limbs, particularly the feet, were most affected—accounting for about 67% of all cases, the head and neck—8·5% of which the eyes were affected in 5·7%. The upper limbs, including the axilla, were the least affected of the sites. Of the 71 melanomas of the lower limbs, 61 (86%) were in the feet, 4 (5·5%) in the legs and 6 (8·5%) were subungual. The preponderance of lesions in the feet duplicated the findings in other pigmented racial groups (Lewis, 1967; Morris and Horn, 1951). The pedal lesions were usually advanced when some of our patients sought medical help (Fig. 7). Intra-ocular melanoma has been reported to be relatively rare in the African (Hogan and Zimmerman, 1962; Macdonald, 1948; Wilder and Paul, 1951, Brown, 1968) in contrast to what obtains in certain European countries where it forms about one-third of all melanomas (Norwegian Cancer Registry, 1961). During a 14-year period in Uganda, there was no recorded case (Davies, *et al.*, 1968), but in the present series, two patients were encountered (Olurin and Williams, unpublished data). Table IV compares the site distribution of melanomas among different population groups. The skin of the trunk, head and neck and upper limbs were less involved in the African or Negro while these sites were more involved in the Caucasians. The lower limbs were particularly highly favoured in the pigmented races (Table IV) and this may be of aetiological significance.

TABLE IV.—*Comparison of Malignant Melanoma in Collected Series*

| | Nigeria | | Morris and Horn U.S. Negro | | Pack *et al.* U.S. White | | Davies *et al.* Uganda African | |
|---|---|---|---|---|---|---|---|---|
| Site | No. | % | No. | % | No. | % | No. | % |
| Lower limbs | 71 | 67·0 | 213 | 79·2 | 366 | 30·4 | 159 | 87·8 |
| Upper limbs | 2 | 1·8 | 13 | 4·8 | 135 | 11·2 | 11 | 5·1 |
| Head and neck | 3 | 2·7 | 11 | 4·1 | 263 | 21·9 | 1 | 0·6 |
| Trunk | 4 | 3·8 | 10 | 3·7 | 291 | 24·2 | 3 | 1·7 |
| Eyes Conjunctiva ... 4, Intraoccular ... 2 | 6 | 5·8 | 28 | 9·8 | 63 | 5·1 | 21 | 10·6 |
| Oral | 1 | 0·9 | 11 | 4·1 | 31 | 2·6 | 6 | 3·3 |
| Anal canal | 3 | 2·7 | 11 | 4·1 | 117 | 9·7 | 1 | 0·6 |
| Others and unspecified sites | 17 | 15·3 | — | — | — | — | — | — |
| Total | 106 | 100% | 287 | 100% | 1235 | 100% | 206 | 100% |

The present series showed a difference between the site incidence of squamous and malignant melanoma (Table II). These differences would tend to point to different aetiological factors in the causation of the two types of malignancy.

During the past ten years, no case of intra-nasal melanoma has been seen in Ibadan. This is in striking contrast to experience in East Africa (Lewis and Martin, 1967) where it is not uncommon. We have, however, seen a few cases of intra-oral melanoma and a case of melanotic ameloblastoma has already been described (Williams, 1967; Akinosi and Williams, 1969).

In the absence of radiotherapy, radical or local excision, amputations and gland dissections were carried out. In some cases, limited surgery combined with cytotoxic therapy were employed. Follow up of about 50 patients showed that about 33% were known to be alive between 1 and 3 years. Six patients who died in the hospital were autopsied while the remaining patients were discharged and presumably died at home. No spontaneous regressions were observed in any of the cases studied by us and there was no clinical indication to suggest that the biological behaviour of these growths differed from what was observed in other races.

The microscopic slides of all the cases were available and they were analysed to ascertain cell types, degree of anaplasia, amount of pigment and where possible, junctional activity. There was no case which can strictly be described as " amelanotic " although there were highly cellular tumours with scanty melanin pigment. The majority of the tumours (64%) were spindle cell or sarcomatous in cell type, some were carcinomatous (26%) and the remainder consisted of both cell types. Metastatic tumour deposits in lymph nodes were usually similar in cell type to the primary tumour although there were a few cases in which the cell morphology differed. Pigment in inguinal lymph nodes of the African is not uncommon and can be attributed to frequent scarification marks and tattooing practices, malarial and schistosomal infections. Fontana's silver stain for melanin and Perl's stain for haemosiderin were usually employed to characterise the pigment.

The relative ratio frequency of malignant melanoma over the 8 year period was 1·6%. When compared with other malignancies encountered in Ibadan, such as of the lungs, cervix, breast, stomach and liver, it was commoner than lung cancer but not as common as the others.

*Ocular Melanomas*

Four cases of conjunctival melanoma and two cases of intraocular melanoma were seen. The intraocular melanomas were histogically confirmed by Dr. Joan Mullaney of the National Ophthalmic Pathology Laboratory of Ireland. All our patients with either conjunctival or intraocular melanomas were in the fourth or fifth decades of life. Of 130 intraocular malignant tumours in our cancer registry over the period of 8 years, only 2 (1·5%) were intraocular melanomas while in the Uganda African no intraocular melanoma was encountered over a 14-year period. However, there are a few isolated reports of intraocular melanoma in the African or Negro in the literature (Brown, 1968).

*Kaposi's Sarcoma*

There were 15 Nigerian patients with Kaposi's sarcoma. All were males, with the typical subcutaneous nodules (Fig. 8). Clinically, conditions which simulate Kaposi's sarcoma include multiple cutaneous neurofibromas and keloid. The youngest patient with Kaposi's sarcoma was 14 and the oldest 72 years old, with an average of 43 years. No albinoes have either been seen here or reported to have Kaposi's sarcoma or keloid. The ages of 11 patients were known and five of these were in the second decade of life, the remaining six were in the fourth and sixth decades. The four patients whose ages were not known were stated to be adults. The majority of the patients had multiple lesions. Only two patients had single lesions, eight had local multiple lesions and six had disseminated multiple lesions.

The regional lymph nodes and other viscera including lungs, liver, adrenals, heart and epididymis were involved with metastases in two patients. The site distribution of the lesions showed that the majority of the lesions occurred on the lower limbs (Table V). A few occurred in the upper limbs and the head and neck, and metastatic lesions were observed in the penis and scrotum in one patient with the disseminated form of the disease. No history of predisposing factors was obtained.

The modes of presentation included oedema of the limbs in seven patients, infected ulcerated nodules in five, pain in four, subcutaneous nodules in four,

TABLE V.—*Site Distribution of 49 Lesions in 15 Patients with Kaposi's Sarcoma*

| Site | Total | % of type |
|---|---|---|
| Lower limbs | | |
| Feet | 10 | 20 |
| Ankles | 8 | 16 |
| Legs | 10 | 20 |
| Thighs | 6 | 12 |
| Upper limbs | | |
| Hands | 4 | 8 |
| Forearm | 4 | 8 |
| Head and neck | | |
| Face | 1 | 2 |
| Scalp | 1 | 2 |
| Neck | 1 | 2 |
| Trunk | 2 | 4 |
| Penis | 1 | 2 |
| Scrotum | 1 | 2 |
| Total | 49 | 100% |

dyspnoea, loss of weight and fever in one each. The histological appearances of the tumours were relatively uniform and characteristic of the late stages since most patients presented late.

Thirteen patients were given intra-arterial nitrogen mustard, and two patients had intravenous mustine hydrochloride. Five patients had excision and graft and cytotoxic drugs, ten patients had cytotoxic drugs only. Of the 13 patients given intra-arterial nitrogen mustard, six had multiple infusions at varying intervals. There was no follow up in seven patients, one patient defaulted, four were known to have survived up to 5 years. Three patients who died from disseminated diseases were aged 19, 42 and 60 years. One patient died from cardiopulmonary failure and two died following the third infusion of mustine hydrochloride. The patients were derived from different parts of the country, but the majority came from the Western and Mid-western States of Nigeria.

The occupations of 14 patients were analysed in order to find out if there was any casual relationship which may lead to identification of any aetiological agent. Six patients were farmers, two were electrical technicians and the others were engaged in clerical, trading and skilled labouring types of occupations. The increased susceptibility of the farmer is noteworthy although its significance is not clear, particularly in an agriculturally orientated country such as Nigeria.

*Albinism and Skin Cancer in Nigeria*

Out of a total of 435 patients with superficial cancers, 15 (3·4%) were complete albinoes, 3 female and 12 male. The 3 females were in their sixth decade and one had squamous carcinoma, another had basal cell carcinoma of skin and the third had malignant melanoma of the anal canal. The patient with anal melanoma had metastatic deposits in regional lymph nodes and in the liver. Local excision was carried out and she was given Endoxan. Eight males had squamous carcinoma, three had basal cell carcinoma and one had sebaceous gland carcinoma. None of the albinoes had cutaneous melanoma. Three albinoes had multiple lesions while the others had single but sometimes extensive lesions. The follow up of these patients was inadequate except in three cases where the lesions recurred several times after removal. When frequencies of lesions in the albinoes were compared with those in pigmented Nigerians, melanomas were rarer in the albino but basal cell carcinomas occurred more frequently. Kaposi's sarcoma and keloids have not been observed in the albino but the significance of this observation is not evident.

### DISCUSSION

It is increasingly useful to study the differences in cancer incidence or frequencies between population groups of different racial and genetic constitution with a view to investigating or identifying factors suspected of carcinogenicity. Data obtained from such studies are usually influenced by uncontrollable factors such as medical care, diagnostic facilities and criteria, selection of patients, location and policies of hospitals and socio-economic levels of the populations.

In developing countries where available demographic data are either not accurate or not available, the ages of the patients are only estimates, histories tend to be unreliable in a significant proportion of patients, and registration of births and deaths are not compulsory, incidence rates are usually difficult to calculate and therefore inaccurate. For this paper, relative ratio frequencies, which have been adopted in other similar reports, are used.

During the 8-year period, 6133 malignant tumours were recorded in our Cancer Registry. Of these, 58 (0·94%) were in the lungs, 127 (2·07%) in the prostate, 303 (4·9%) in the breast, 438 (7·1%) in the liver and 618 (10·7%) in the cervix. The relative ratio frequency of superficial cancers of this series, over the 8-year period, was 7·2%. The individual frequencies for the four main types of malignancy considered, namely squamous carcinoma, malignant melanoma, basal cell carcinoma and Kaposi's sarcoma were approximately 4·8, 1·6, 0·4 and 0·5% respectively. It was evident that superficial cancers had the same relative ratio frequency as liver cancer but occurred less frequently than cervical cancer. Squamous carcinomas alone (4·8%) had the same frequency as breast cancer (4·9%) but occurred more frequently than prostatic (2·07%) and lung cancers (0·94%). It would therefore appear that superficial cancers have a relatively high ratio frequency in Nigeria. Although Smith and Elmes (1934), in a previous report from Nigeria, gave a higher figure of 15%, Edington and Maclean (1965), in a 3-year cancer rate survey reported a relative ratio frequency of 6·4%. There is conflicting evidence about the incidence or frequency of skin cancer in pigmented races including Africans (Vint, 1935; Gelfand, 1949; Geyer, 1947). Shapiro and his colleagues (1953) found 50 (8·4%) cases of skin cancer out of 590 cases of malignant disease in South African Bantus over a period of three years and concluded that it was a rare condition. Steiner (1954) found only one American Negro with carcinoma of the scrotum in Los Angeles out of 135 patients and Schrek (1944) found that skin cancer accounted for only 3% of tumours in American Negroes (male military personnel). Davies and his colleagues (1968) found that superficial cancers of the skin constituted up to 15% of all cancers diagnosed among the Uganda Africans and in a survey carried out by the National Cancer Institute of U.S.A. in 1947–48, the crude incidence rate of skin cancer in the non-whites was about one-sixth to one-twentieth that of the whites. Low ratio frequencies have also been obtained in other pigmented population groups in India (Khanolkar, 1950), Indonesia (Kouwenaar and Sutomo, 1951), North Africans (Mussini and Montpellier, 1951),

West Indian Negroes (Tomlinson and Wilson, 1945) and Cubans (Puente-Duany, 1951).

There are, however, certain findings which are common to these reports and confirmed by the present study. One of these is that the frequency of basal cell carcinoma in the pigmented races is lower than in Caucasians. In the present series, 21 patients (0·4%) had basal cell carcinomas out of whom six were albinoes. The high frequency of albinoes in this and other series and the fact that the lesion is commoner in Europeans tend to suggest that absent or diminished skin pigmentation tends to predispose to or facilitate the growth of this type of tumour. It is noteworthy that basal cell carcinomas in pigmented races not only arise on the face as they usually do in the Causcasian but may be situated in unusual sites such as trunk, perineum and the lower limbs.

Although comparison of true incidences cannot be made between the American Negroes and West Africans, it appears that the latter has a higher relative ratio frequency of superficial cancers. This is an example of a neoplasm which may have changed following migration due, not to genetic factors, but to environmental factors. The observed differences in frequencies of superficial cancers in the different racial groups may also be due to variation in environmental factors conditioned by degree of skin pigmentation.

In the case of malignant melanoma, a previous observation which is confirmed in this study is the relative rarity of intraocular melanomas in the African. In some series, conjunctival melanomas in the African are not uncommon but intraocular melanomas are rare. The rarity may either be real or be a reflection of ophthalmological services in the communities. The latter is thought to be unlikely, particularly in medical teaching centres in Africa. Another feature common to pigmented races is the site distribution of cutaneous melanoma. While the majority of melanomas occur in the lower extremities of the negro or bare-footed African, they almost invariably occur in the upper extremities of the Caucasian. This difference can be attributed to possible aetiological factors such as trauma (Hewer, 1935). Kaposi's sarcoma in Nigeria has been discussed in other papers (Maclean, 1963; Oluwasanmi and Osunkoya, 1969) and will not be discussed here in detail.

Owing to the absence of radiotherapy in several centres in Africa and the late presentation of superficial cancers, the prognosis is invariably poorer than in other developed countries.

### SUMMARY

This paper analyses 435 cases of superficial cancers in Nigerians over a period of eight years. Superficial cancers accounted for about 7·2% of all tumours registered in the Cancer Registry. The most common tumour encountered was squamous carcinoma which accounted for about 67% of all superficial cancers, followed by malignant melanoma which accounted for about 24% of all superficial cancers. Basal cell carcinomas were relatively rare (4·8%) and the low frequency was consistent with reports in other pigmented races. Kaposi's sarcoma accounted for 3·4% of all superficial cancers. The clinico-pathological features of these tumours are discussed. It is suggested that variations in relative frequencies of the various superficial cancers in different races may be attributed to different environmental factors conditioned by degree of skin pigmentation.

We are grateful to the British Empire Cancer Campaign for Research for the support of the Cancer Registry and to Mrs. Margaret Hendrickse of the Cancer Registry for her invaluable help with the records. We wish to thank the staff of the Medical Illustration Unit for the photographs.

### REFERENCES

AKINOSI, J. O. AND WILLIAMS, A. O.—(1969) *Oral Surg.*, **27**, 257.
BROWN, I. A. R.—(1968) *Br. J. Ophthal.*, **52**, 184.
COHEN, L., SHAPIRO, M. P., KEEN, P. AND HENNING, A. J. H.—(1952) *S. Afr. med. J.*, **26**, 932.
DAVIES, J. N. P., KNOWELDEN, J. AND WILSON, B. A.—(1965) *J. natn. Cancer Inst.*, **35**, 789.
DAVIES, J. N. P., TANK, R., MEYER, R. AND THURSTON, P.—(1968). *J. natn. Cancer Inst.*, **41**, 31.
EDINGTON, G. M. AND EASMON, C. O.—(1967). *Natn. Cancer Inst. Monogr.* No. 25, p. 8.
EDINGTON, G. M. AND MACLEAN, C. M. U.—(1965) *Br. J. Cancer*, **19**, 470.
GELFAND, M.—(1949) *S. Afr. med. J.*, **23**, 1010.
GEYER, A.—(1947) *Bull. Soc. Path. exot.*, **40**, 125.
HEWER, T. F.—(1935) *J. Path. Bact.*, **41**, 473.
HIGGINSON, J. AND OETTLÉ, A. G.—(1960) *J. natn. Cancer Inst.*, **24**, 589.
HOGAN, M. J. AND ZIMMERMAN, L. E.—(1962) 'Ophthalmic Pathology'. Philadelphia (Saunders).
HUTT, M. S. R. AND BURKITT, D. P.—(1965) *Br. med. J.*, **2**, 719.
JANOTA, I.—(1967) *Br. J. Derm.*, **79**, 5, 259.
KHANOLKAR, V. R.—(1950) *Acta Un. int. Cancer.*, **6**, 881.
KOUWENAAR, W. AND SUTOMO, T.—(1951) *Acta Un. int. Cancr.*, **7**, 61.
LEWIS, M. G.—(1967) *Br. J. Cancer*, **21**, 483.
LEWIS, M. G. AND MARTIN, J. A. M.—(1967) *Cancer, N.Y.*, **20**, 1699.
MACDONALD, E. J.—(1948) *Spec. Publs N.Y. Acad. Sci.*, **4**, 71.
MACLEAN, C. M. U.—(1963) *Br. J. Cancer*, **17**, 195.
MARSHALL, J.—(1964) ' Skin diseases in Africa'. Cape Town (Maskew Miller).
MORRIS, G. C., JR. AND HORN, R. C., JR.—(1951) *Surgery, St. Louis*, **29**, 223.
MUSSINI-MONTPELLIER, J.—(1951) *Acta. Un. int. Cancr.*, **7**, 77.
NORWEGIAN CANCER SOCIETY—(1961) ' Cancer Registration in Norway. The incidence of Cancer in Norway, 1953–1958 ', Oslo.
NTSEYABALIWE, F. W. AND MLUGE, G.—(1965) *Makerere med. J.*, **8**, 1965.
OLUWASANMI, J. O. AND OSUNKOYA, B. O.—(1969) *W. Afr. med. J.*, **18**, 89.
PACK, G. T., LENSON, N. AND GERRER, D. M.—(1952) *A.M.A. Archs Surg.*, **65**, 862.
PRATES, M. D. AND TORRES, F. D.—(1965) *J. natn. Cancer Inst.*, **35**, 729.
PUENTE DUANY, N.—(1951) *Archos cub. cancer.*, **10**, 225.
SCHREK, R.—(1944) *Cancer Res.*, **4**, 119.
SHAPIRO, M. P., KEEN, P., COHEN, L. AND MURRAY, J. F.—(1953) *Br. J. Cancer*, **7**, 45.
SMITH, E. L. AND ELMES, B. G. T.—(1934) *Ann. trop. med. Parasit.* **28**, 461.
STEINER, P. E.—(1954) ' Cancer: Race and geography '. Baltimore (Williams and Wilkins Co.).
TOMLINSON, W. J. AND WILSON, L. A.—(1945) *Cancer Res.*, **5**, 368.
VINT, F. W.—(1935) *Lancet*, ii, 628.
WILDER, H. C. AND PAUL, E. V.—(1951) *Milit. Surg.*, **109**, 370.
WILLIAMS, A. O.—(1967) *J. Path. Bact.*, **93**, 545.
WILLIAMS, A. O. AND EDINGTON, G. M.—(1967) *Dis. Colon Rectum*, **10**, 301.